# Complex Effects of Ecosystem Engineer Loss on Benthic Ecosystem Response to Detrital Macroalgae

**DOI:** 10.1371/journal.pone.0066650

**Published:** 2013-06-21

**Authors:** Francesca Rossi, Britta Gribsholt, Frederic Gazeau, Valentina Di Santo, Jack J. Middelburg

**Affiliations:** 1 Laboratoire Ecologie des Systèmes marins côtiers, Université Montpellier 2, Montpellier, France; 2 Department of Ecosystems, Royal Netherlands Institute for Sea Research, Yerseke, the Netherlands; 3 Centre National de la Recherche Scientifique-Institut National des Sciences de l’Univers, Laboratoire d’ Oceanographie de Villefranche, Villefranche-sur-mer, France; 4 Université Pierre et Marie Curie, Observatoire Océanologique de Villefranche, Villefranche-sur-mer, France; 5 Department of Biology, Boston University, Boston, Massachusetts, United States of America; 6 Department of Earth Sciences – Geochemistry, Faculty of Geosciences, Utrecht University, Utrecht, The Netherlands; Bangor University, United Kingdom

## Abstract

Ecosystem engineers change abiotic conditions, community assembly and ecosystem functioning. Consequently, their loss may modify thresholds of ecosystem response to disturbance and undermine ecosystem stability. This study investigates how loss of the bioturbating lugworm *Arenicola marina* modifies the response to macroalgal detrital enrichment of sediment biogeochemical properties, microphytobenthos and macrofauna assemblages. A field manipulative experiment was done on an intertidal sandflat (Oosterschelde estuary, The Netherlands). Lugworms were deliberately excluded from 1× m sediment plots and different amounts of detrital *Ulva* (0, 200 or 600 g Wet Weight) were added twice. Sediment biogeochemistry changes were evaluated through benthic respiration, sediment organic carbon content and porewater inorganic carbon as well as detrital macroalgae remaining in the sediment one month after enrichment. Microalgal biomass and macrofauna composition were measured at the same time. Macroalgal carbon mineralization and transfer to the benthic consumers were also investigated during decomposition at low enrichment level (200 g WW). The interaction between lugworm exclusion and detrital enrichment did not modify sediment organic carbon or benthic respiration. Weak but significant changes were instead found for porewater inorganic carbon and microalgal biomass. Lugworm exclusion caused an increase of porewater carbon and a decrease of microalgal biomass, while detrital enrichment drove these values back to values typical of lugworm-dominated sediments. Lugworm exclusion also decreased the amount of macroalgae remaining into the sediment and accelerated detrital carbon mineralization and CO_2_ release to the water column. Eventually, the interaction between lugworm exclusion and detrital enrichment affected macrofauna abundance and diversity, which collapsed at high level of enrichment only when the lugworms were present. This study reveals that in nature the role of this ecosystem engineer may be variable and sometimes have no or even negative effects on stability, conversely to what it should be expected based on current research knowledge.

## Introduction

It is widely recognized that species loss may affect ecosystem stability and, in turn, have important social and ecological consequences [1]–[3]. Overall, when more species are present in a community, ecosystem functions are more stable in time and increase their resistance or resilience against disturbance, due to an increased variety of life strategies, functional traits and responses to environmental disturbance [4]–[6]. Different species contributing to similar ecosystem functions can occur under different environmental conditions and ensure that functions are performed even when some other species are displaced [4], [5], [7].

Empirical evidence has shown that sometimes species loss can have complex, often idiosyncratic effects on the stability of ecosystem functions and on both their resistance and resilience against disturbance [8], [9]. This is especially true for the marine coastal ecosystem, where species identity, functional traits and environmental context may rule ecosystem functions and their response to disturbances [10]–[12]. One or few species can be key contributors to ecosystem functions to the extent that their loss can outweigh the effect of species richness on ecosystem functions, resilience or resistance [10], [13], [14]. Such overwhelming effect can, however, vary with the environmental context and change with increasing ecological complexity [15]. It seems thus evident that understanding the role of species loss in marine systems might require a large body of knowledge of the functional role of individual species and of the consequences of losing particular species for ecosystem functions and for their response to disturbance.

Ecosystem engineer species strongly modify resource availability and environmental conditions, which in turn can alter communities and ecosystem functions [16], [17]. These species are thus ideal candidates to test hypotheses on how local extinction of key contributors to ecosystem functions can modulate the response of ecosystems to disturbance. In marine sediments and terrestrial soils, ecosystem engineers are often species that contribute to particle mixing and solute penetration into the sediment through the process of bioturbation, which includes sediment reworking and burrow ventilation [18]. These bioturbating engineer species can be great contributors to diagenetic reactions, to the recycling of sedimentary organic matter, and to benthic community structure and biodiversity [18]–[21]. They also have the potential to strongly modify the response to disturbance of benthic communities and functions [22]. For instance bioturbation by *Marenzellaria viridis* has been found to decrease hypoxic crises in the Baltic Sea [23]. It has also been found that in soil, reworking activity of dominant contributors to bioturbation, such as earthworms, modulates the response of plant communities to invasions [24]. Surprisingly, however, ecosystem engineer loss has been poorly considered in studies investigating ecosystem response to disturbance.

In marine coastal systems, a large portion of seaweeds and seagrasses is not consumed by herbivores but returns to the environment as decaying organic matter [25]. In shallow-water coastal habitats, detrital seaweeds and seagrasses can supply important amounts of organic matter to benthic ecosystems. They represent an important source of disturbance because during decomposition they can alter sediment biogeochemistry and, in turn, benthic communities of consumers. Despite detrital seaweed and seagrass supply is often a natural event, proliferation and blooms of seaweeds as a consequence of eutrophication can dramatically increase detrital seaweed biomass and exacerbate the effects on benthic ecosystems [26]–[29]. Biomass decomposition can induce hypoxia followed by the release of reduced toxic compounds such as sulphides, which are toxic for several benthic species and can therefore strongly reduce species abundance and diversity [26], [27], [29]–[33]. Detrital decomposition can also provide food, directly for detritivores or indirectly, by stimulating bacterial metabolism, thereby altering the benthic food web [28], [34]. These effects strongly depend on the amount of detrital inputs and on its decomposition patterns, which may strongly vary as a consequence of benthic invertebrate composition and identity [35].

Bioturbating fauna can modulate the recycling of detrital macroalgae enhancing organic matter mineralization and microbial activity [36]–[38]. Some burrowing bioturbating species, such as certain crabs or polychaetes, can also incorporate detritus into their burrow wall lining and represent important sinks for detrital macroalgae [39]. In addition, often bioturbating infauna are also detritivores that can consume directly detritus and contribute to its recycling in the food web. In a laboratory experiment, for instance, it was found that nitrogen and carbon fluxes at the sediment-water interface changed following both the addition of the detrital green macroalgae *Chaetomorpha linum* and the presence of the bioturbating polychaete *Nereis diversicolor.* This polychaete ingested macroalgal fragments, enhanced microbial metabolism and contributed to diffuse solutes in the interstitial sediment and at the sediment-water interface [36].

One common well-recognized bioturbating species typical of temperate waters is the burrowing lugworm *Arenicola marina.* This worm lives head down in J-shaped burrows where it ingests sediment and defecates on the surface. Its activity has been recognized to destabilize sediment and counteract the effect of microphytobenthos, increase sediment microhabitats and alter sediment biogeochemistry, by enhancing solute penetration into sediment depth and re-distributing organic material along the vertical profile [40]. Consequently, this species may alter other fauna and vegetation [36], [37], [41]. This study reports on how the loss of this bioturbating lugworm may alter the response to detrital macroalgae of intertidal benthic macrofauna and sediment biogeochemistry. It was hypothesized that the deliberate exclusion of lugworms would increase the magnitude of changes caused by the disturbance of macroalgal detritus for (i) carbon availability and benthic respiration; (ii) microalgal biomass and (iii) macrofauna assemblages. Recycling of the organic carbon derived from detrital decomposition was also investigated to explore some of the mechanisms that might regulate the effect of *A. marina* on benthic responses to macroalgal detritus.

## Materials and Methods

### Study Site and Experimental Design

**Figure 1:**
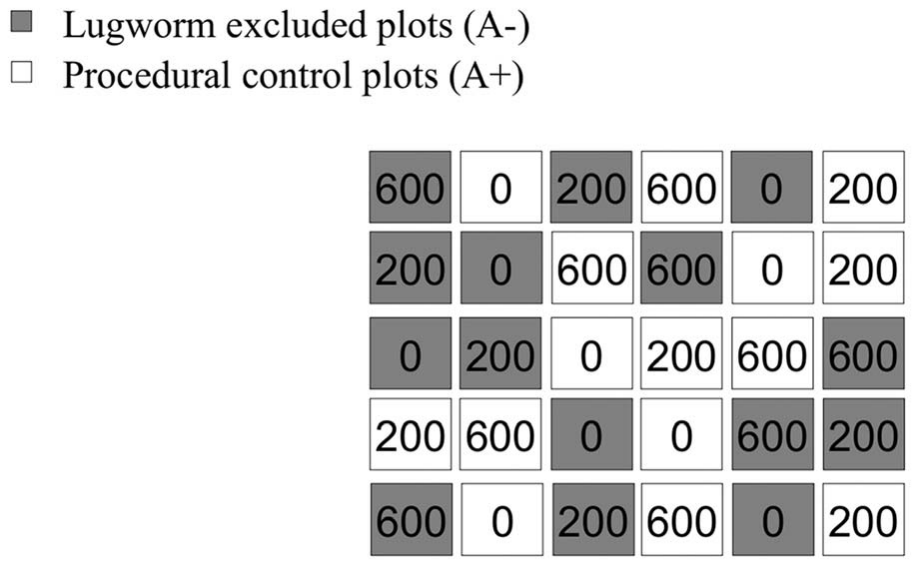
Schematic experimental design for experiment 1. Each rectangle corresponds to 1 m^2^ plot. Numbers refers to the detrital enrichment. (0: no addition; 200∶200 g WW were added twice at a 1 week interval; 600∶600 g WW were added twice with a 1 week interval). Distance among plots are not in scale.

Two manipulative experiments were done on an intertidal flat of the Oosterschelde estuary (The Netherlands) dominated by the bioturbating lugworm *Arenicola marina,* during summer 2006. The study intertidal flat, named Slikken van Viane (51° 37′ 00′′ N, 4° 01′ 00′′ E), was a sheltered area situated on the upper intertidal close to a salt marsh. Sediment grain-size was 65% fine sand and 35% very fine sand (Rossi F., unpublished data). The Oosterschelde estuary is a national park designated as zone VI by the International Union for Conservation of Nature (IUCN). This IUCN classification has among its objectives the facilitation of scientific research and environmental monitoring, mainly related to the conservation and sustainable use of natural resources (http://www.iucn.org). No specific permits were therefore required for performing these scientific experiments, which did not involve endangered or protected species. The first experiment tested the effect of *A. marina* exclusion on the response of macrofauna assemblages and sediment biogeochemistry to different amounts of detrital macroalgae. The second experiment measured the changes in detrital recycling following *A. marina* exclusion. For the first experiment (Fig. 1), all lugworms were excluded from 15 randomly chosen sediment plots of 1×1 m, some meters apart, by burying mosquito nets to the depth of approximately 7 cm (hereafter exclusion A− treatment), following the method proposed by [41] in a more extensive area. A procedural control for the effect of the net and of the burial was done for other 15 sediment plots of 1×1 m by burying mosquito nets with 10×10 cm holes (hereafter procedural control A+ treatment).

One month after lugworm exclusion, the sediment plots were organically-enriched by addition of detrital *Ulva* spp. Fresh *Ulva* thalli were previously collected in the Oosterschelde estuary and washed to remove animals and sediment. Thalli were then weighted and frozen in separate plastic bags. Freezing is commonly used in experiments that aims at testing effects of detrital macroalgae and decomposition patterns. This treatment is necessary in order to create detritus and ensure that *Ulva* is dead when buried since this seaweed can survive for very long periods in the dark [30]. The thalli were left defrosting at air temperature and then added to the top 1 cm of the sediment by gently hand-churning the detritus with the sediment. Enrichment was done twice, on July 3 and July 10, 2006. Each time either 200 or 600 g of wet weighted *Ulva* (hereafter treatments 200 and 600, respectively) were added to 10 exclusion (A−) and 10 procedural control (A−) plots. These amounts corresponded to 40 and 120 g dry weight. Other 10 plots (5 A− and 5 A+) were not enriched (hereafter treatment 0), but the sediment was hand-churned to control for the manipulation. There was no procedural control for the physical presence of detritus as previous experiments had demonstrated no effects [28]–[30]. The amount of *Ulva* spp. was based on the available literature to cause medium and high levels of disturbance (e.g. [27], [30], [32], [33]).

The second experiment was run at the same time of experiment 1. Two additional procedural control plots for burial and 2 exclusion plots were enriched with 200 g ^13^C-labelled *Ulva* twice, resembling the treatment “200”. These four plots were used to trace the fate of the carbon released from detrital *Ulva*. The labeling of *Ulva* was done in the laboratory. Leaves were washed and carefully cleaned to eliminate epiphytes and animals. Then, thalli were transferred to aquaria containing filtered seawater supplemented with NaH^13^CO_3_ (99% ^13^C) to the concentration of 0.2 g L^−1^. The aquaria were oxygenated and covered with transparent PVC to limit ^13^C bacterial respiration.

### Field Sampling

For the first experiment, sampling was done on July 30 and August 1, 2006, one month after detrital enrichment. This timing was chosen because previous studies suggested that effects of algal mats on macrofauna occur within 2 to 10 weeks from the deposition [31]–[33], [42] and that decomposition of *Ulva* occurs within a month (e.g. [43], [44]).

Sediment samples were taken for organic carbon (hereafter OC), porewater total inorganic carbon (DIC = H2CO_3_+ HCO_3_^−^+CO_3_^2−^), microalgal biomass (chlorophyll *a*) and macrofauna species composition, abundance and diversity. The sediment for chlorophyll *a* and OC was collected using a cut syringe of 3 mm inner diameter (i.d.). For chlorophyll *a*, the sediment was collected to 1 cm depth, kept in the dark and preserved at −80°C. The sediment for OC was collected to the depth of 6 cm, sectioned into 0–1, 1–2 and 2–6 cm depths and preserved freeze-dried. *Ulva* detritus was removed from this sediment under the stereo-microscope. The sediment for porewater DIC extraction was collected with cores of 5 cm i.d. and sectioned into 0–1, 1–2 and 2–6 cm depth intervals at the lowest and highest levels of organic enrichment (0 and 600) in both the control and the exclusion treatments. Two cores were collected from each plot and then pooled to reach the amount of water needed for the analyses. Porewater was extracted by centrifugation (1344 g, 15 min) and the supernatant was filtered on Millipore 45 µm. The extracted water was stored in glass vials, sealed, immediately poisoned with saturated HgCl_2_ to stop bacterial activity and analyzed within 3 d. Only 3 out of 5 randomly-chosen replicate plots were sampled for OC and TCO_2_.

The sediment for macrofauna was collected using 11 cm i.d. PVC core. This sediment was sieved (0.5 mm mesh) and macrofauna fixed in 4% buffered formol. Before sampling, all plots were photographed to estimate the abundance of *A. marina* using visible mounds. In the laboratory, all plots were independently examined by four researchers to reduce estimate bias. Five randomly-chosen natural plots were also photographed and then sampled using 11 cm i.d. cores to test for the effect of the procedural control A+ on the natural abundance of *A. marina* and of the other benthic macrofauna. No differences were detected between natural and procedural control treatments (see Results section) and this latter was used for evaluating the effect of detrital enrichment and lugworm exclusion.

Sediment cores were also used to measure fluxes of inorganic carbon (TCO_2_) and oxygen (O_2_) at the sediment-water interface. Intact sediment cores were collected by pushing Plexiglas cores (11 cm i.d.) into the sediment to a depth of 20 cm at each of three replicate plots for the lowest and the highest levels of organic enrichment (0 and 600) in both the control and the exclusion treatments. The net was carefully cut around the cores to allow the core penetration into the sediment. Cores were sealed with rubber stoppers and dug out of the sediment. All cores were brought to a climate controlled room within 2 h, and kept in the dark at *in situ* temperature (10°C) before further handling. The same day, the cores were incubated in the dark for 12 h. Incubation in the dark did not allow to gather additional information concerning the (auto)trophic state of the sediment, which would need a light incubations of other sediment cores. Our system was however limited to carry 12 cores at a time and no further analyses could be done. Prior to incubations, cores were inundated with well-oxygenated Oosterschelde water collected the same day ([NH_4_^+^] = 20 µmol L^-^, [NO_3_^−^] = 110 µmol L^−1^, salinity = 22) and left to acclimatize in the dark for 6–8 h. During incubation, the overlying water-column (25–30 cm) was continuously stirred with a magnetic stirrer, maintaining continuous water circulation at a rate well below the resuspension limit. Dissolved O_2_ never decreased below 65% of saturation. Water samples for TCO_2_ and O_2_ were collected in glass vials and sealed avoiding air bubbles. All samples were immediately poisoned with saturated HgCl_2_ to stop bacterial activity and then analyzed within 3 d.

For experiment 2, sediment samples were taken from the four plots with labelled *Ulva.* Sampling was done on four occasions, 6, 8, 15 and 17 days after detrital enrichment. Each time, one plexiglass intact sediment core (11 cm i.d.) was collected to 20 cm depth, following the modality used to sample the cores for estimating benthic respiration. To reduce plot damage during core collection, the central part of the plot (20 cm from the edges) was divided into 4 quadrants of 30×30 cm. Each sampling occasion, a sediment core was taken from a different quadrant. An area of 15×15 cm was thus damages each time, corresponding to 2.25% of the entire plot area. The sampled sediment was replaced with plexiglass tubes to avoid any collapse and to leave the remaining part of the plots undisturbed. The cores were handled like the cores used for evaluating oxygen and carbon exchange at the sediment-water interface for the experiment 1. However, before incubating for wet respiration and determining release of T^13^CO_2_ in the water column, the labelled cores were incubated for dry fluxes during 6 h. At the beginning and at the end of the incubation, air samples were collected using syringes and pumped into tubes containing soda lime to trap CO_2_ and measure the ^13^CO_2_ derived from *Ulva* decomposition. A LICOR automated CO_2_ analyzer was used to quantify CO_2_ release every hour. Sediment-water exchange rate and macrofauna data were generated as for experiment 1 (see “Analytical measures” section).

Following flux incubations for both experiments, the cores were sieved at 0.5 mm to collect the macrofauna. No lugworm was included in the cores, meaning that any observed pattern represented the result of sediment and macrofauna alteration due to *Arenicola*, but not the direct activity of the lugworm during incubation.

### Analytical Measures

In the laboratory, macrofauna were identified to the species level, counted and dried at 60°C for 48 h to obtain dry weight biomass to be used for stable isotope analyses relative to the experiment 2. The detrital *Ulva* remaining in the samples was also collected, dried and weighed. Chlorophyll *a* was extracted in darkness for 24 h at 0–4°C using a 90% acetone solution. The sediment was homogenized and sub-samples of about 1 g dry weight were taken. Pigments were measured spectrophotometrically before and after 0.1 N HCl acidification. Pre-combusted (450°C for 3 h) sediment was used as a blank. Measurements for bulk sediment OC were made using a Perkin-Elmer CHN element analyzer on freeze-dried material, after carbonate removal with HCl.

Flux rates of TCO_2_ and O_2_ between the sediment and the overlying water were estimated as difference between the initial and the final TCO_2_ or O_2_ concentrations during dark incubation, assuming constant solute exchange with time. The oxygen dissolved into the water samples was analyzed using the standard method of Winkler titration, while water TCO_2_ and porewater DIC were determined by the flow injection/diffusion cell technique, which is based on the diffusion of CO_2_ through a hydrophobic membrane into a flow of deionized water, thus generating a gradient of conductivity proportional to the concentration of CO_2_.

Water TCO_2_ (wet fluxes), gaseous CO_2_ (dry flux), bulk sediment and macrofauna collected in the labelled plots were analyzed for carbon isotope composition (δ^13^C). The gaseous ^13^CO_2_ (dry flux) captured using soda lime was analyzed after acidification. The ^13^C isotope signature for TCO_2_ dry and wet fluxes was analyzed using a GC column coupled to a Finningan delta S mass spectrometer IRMS. The carbon isotopic composition for sediment, residual detritus and animals was determined using a Fisons elemental analyzer coupled to a Finningan delta S mass spectrometer. ^13^C to ^12^C ratio was referred to Vienna PDB and expressed in units of ‰. Reproducibility of the measurements is better than 0.2‰ [45]. The excess of the heavy isotope of carbon (above background) was expressed as total uptake (I) in milligrams of ^13^C. I was calculated as the product of excess ^13^C (E) and carbon or biomass (C):
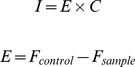
where F indicates the fraction of isotope added and it is calculated as:



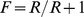
where R can be calculated from the measured δ^13^C using R _reference_ of 0.0112372:



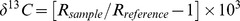



### Statistical Analyses

For experiment 1, data were analyzed with two-way orthogonal mixed model of analysis of variance (ANOVA), with exclusion (2 levels: procedural control A+ and exclusion of *A. marina,* A−) and detrital enrichment (3 or 2 levels: 0, 200 and 600) as fixed and random factors, respectively. Detrital enrichment was considered a random factor because the amount of detritus to add was chosen within a range of values to simulate conditions of low and high enrichment, but no specific hypothesis concerned the chosen amounts. A significant interaction between exclusion and detrital enrichment was expected if the exclusion of *A. marina* changed the response to detrital enrichment. These analyses were done for the total abundance of macrofauna individuals, number of macrofauna species, chlorophyll *a*, sediment OC, porewater DIC, water O_2_ and TCO_2_ exchange rate. *C-cochran* test was used to test for residual variance homogeneity before running the analyses. When this test was significant (*P*<0.05), data were transformed. Pooling was done when *P*>0.25. *A posteriori* multiple comparison SNK test was run when interaction term was significant.

The variables collected for evaluating the recycling of detrital ^13^C (experiment 2) were analyzed with repeated measure ANOVA, with time (6, 8, 15 and 17 sampling days from the end of detrital addition) as the repeated measure random factor and *A. marina* exclusion as fixed factor. Plots (2 levels) was nested in exclusion. The interaction of time with the exclusion treatment was measured over the interaction time×plot. When significant interaction was found, the main effect exclusion was not interpreted. Assumption about the correlation among the different observations of the repeated measure factor (time) was tested with Mauchley’s sphericity test.

Decomposition rate of macroalgal detritus was estimated by fitting the first-order model:
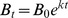
Where B_t_ is the g of detrital biomass at time t (days), B_0_ is the initial biomass, at time 0, at the moment of the second detrital addition and k is the specific decomposition constant. The two detrital additions were done at a distance of one week, which may greatly affect the estimates of B_0_. We therefore considered the estimates of residual *Ulva* as the sum of two decomposition curves, with the same constant k but different decomposing time:



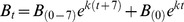



The curves of ^13^C benthic incorporation (including loss due to respiration) were fitted to the logistic curve:
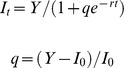
Where Y is the ^13^C loss from macroalgal decomposition and r is the capacity of benthos to incorporate ^13^C.

## Results

**Figure 2:**
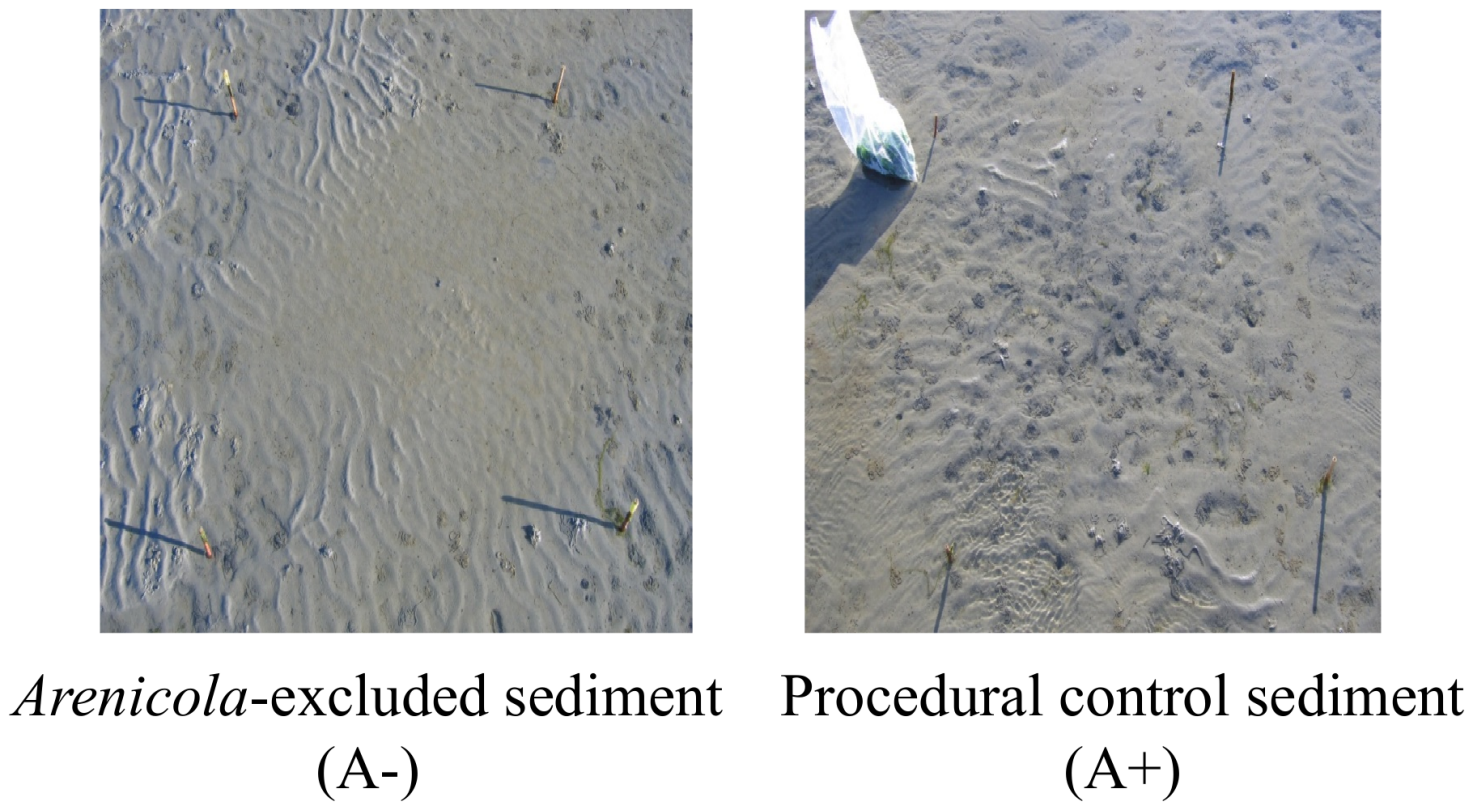
Comparison of sediment surface appearance after *Arenicola marina* exclusion at low tide. A− represent the sediment after 1 month from the burial of net to exclude burrowing species. A+ is the procedural control treatment (see Materials and Methods for details).

After the exclusion of *A. marina*, no burrows were visible in the excluded plots (Fig. 2) and the number of burrows in the procedural control (treatment A+) was comparable to that of natural sediment (mean±SE, n = 5∶8.72±1.77 and 10.33±1.17 burrows m^−2^ for the natural and the procedural control, respectively). Overall, there were no differences in macrofauna species composition between the natural sediment and the procedural control plots. Macrofauna abundance (mean±SE, n = 5) was 294±42 and 220±53 individual m^2^, whereas the number of species was 10±0.2 and 10±0.5 for A+ and natural sediment, respectively. Therefore, A+ could be used as a control for the effect of lugworm exclusion and detrital macroalgae.

Overall, in the natural sediments and in the A+ plots the gastropod *Hydrobia ulvae* was numerically dominant, representing 90% of macrofauna abundance. The remaining 10% was made up by oligochaetes (3%), deposit feeder polychaetes (3%) such as spionids, nereids, capitellids and cirratulids and bivalves (3%), mainly *Macoma balthica* and *Cerastoderma edule*.

### Carbon Availability and Benthic Respiration

**Figure 3:**
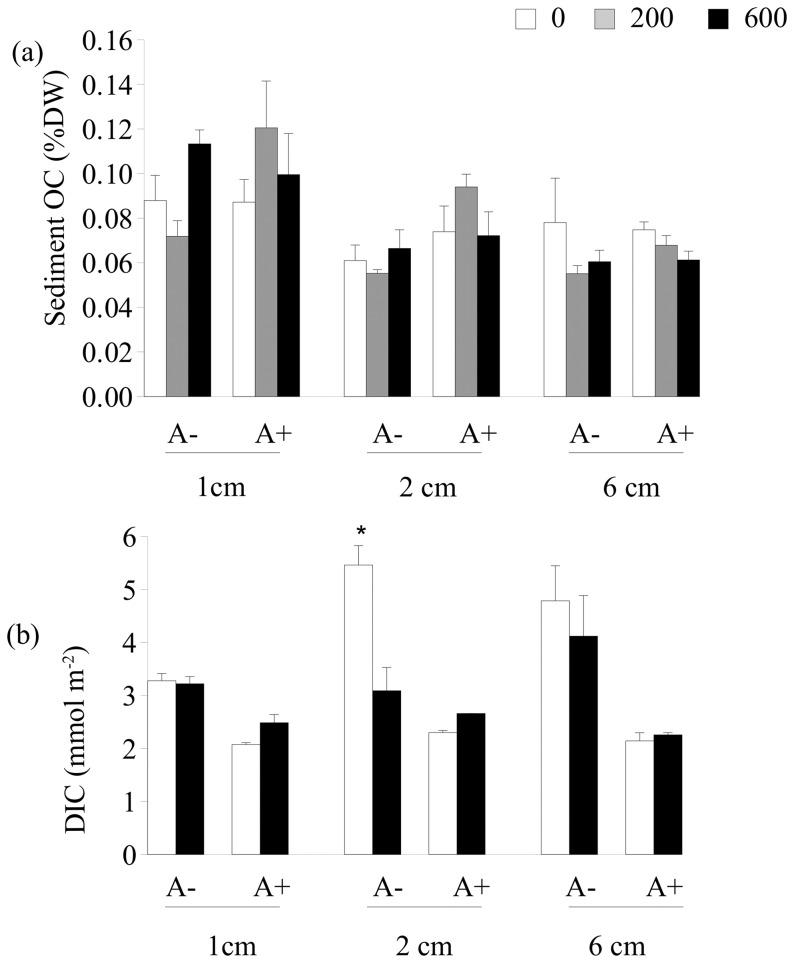
Mean (+1SE) for (a) bulk sediment organic carbon (OC) and (b) porewater dissolved inorganic carbon (DIC) at different sediment depths (*1 cm*: surface sediment to 1 cm depth; *2 cm*: from the depth of 1 to 2 cm; *6 cm:* from the depth of 2 to 6 cm). In the graphs, A−: *A. marina* excluded; A+: procedural control for burial; the legend indicate the levels of detrital addition (0: no addition; 200∶200 g WW were added twice at a 1 week interval; 600∶600 g WW were added twice with a 1 week interval). Data were sampled one month after detrital addition. Asterisk indicates significant differences.

At the time of sampling, sediment organic carbon (OC) did not vary following detrital enrichment, the exclusion of *A. marina* or their interactions (Table 1, Fig. 3a; ANOVA, *P*>0.05). Macroalgal debris was still present in the sediment where detrital macroalgae were added at the highest enrichment level (treatment 600). The biomass of this macroalgal debris was smaller in the lugworm-excluded sediment than in procedural controls (mean±SE: lugworm exclusion, treatment A−: 2.3±1.4 gDW m^−2^; A+: 12.0±6.6 gDW m^−2^; ANOVA: *F*_1, 8_ = 6.19, *P* = 0.038).

**Table 1:** Two-way analyses of variance for sediment organic carbon (OC) and porewater DIC at different sediment depths one month after detrital enrichment.

		0–1 cm		1–2 cm		2–6 cm	
Sediment OC	df	MS	F	MS	F	MS	F
Exclusion = E	1	0.67 10^−3^	0.40	1.60 10^−3^	3.80	0.09 10^−3^	0.52
Detrital enrichment = D	2	0.61 10^−3^	0.96	0.09 10^−3^	0.40	0.62 10^−3^	2.22
E×D	2	1.7 10^−3^	2.64	0.42 10^−3^	1.90	0.17 10^−3^	0.62
Residual	12	0.63 10^−3^		0.22 10^−3^		0.28 10^−3^	
**Porewater DIC**							
Exclusion = E	1	1.89	17.50	5.06	1.03	10.15	4.80
Detrital enrichment = D	1	0.06	2.00	1.28	5.12	0.15	0.28
E×D	1	0.11	3.43	4.93	**19.71**	0.30	0.57*
Residual	8	0.03		1.00		2.11	

Significant values (*P*<0.05) are in bold. Data were not-transformed (*P*_C-cochran_ >0.05). Asterisk indicates pooling of interaction when *P*>0.25.

There was a significant effect of the interaction between lugworm exclusion and detrital enrichment on porewater DIC to the depth of 1–2 cm (Table 1; Fig. 3b). In the non-enriched sediment, porewater DIC concentration was higher in the lugworm exclusion than in the control plots (SNK test at *P* = 0.05: non-enriched plots, treatment 0: A−>A+). Moreover, in the lugworm-exclusion plots, porewater DIC was lower in the enriched than in the non-enriched plots (SNK test at *P* = 0.05: *Arenicola* exclusion, A−: 0>600). Values in detrital-enriched, lugworm-excluded sediment were similar to those measured for the A+ treatment (Fig. 3b). A similar pattern was observed for superficial (0–1 cm) and deeper sediment (2–6 cm in Fig. 3b), but no significant changes were measured.

**Figure 4:**
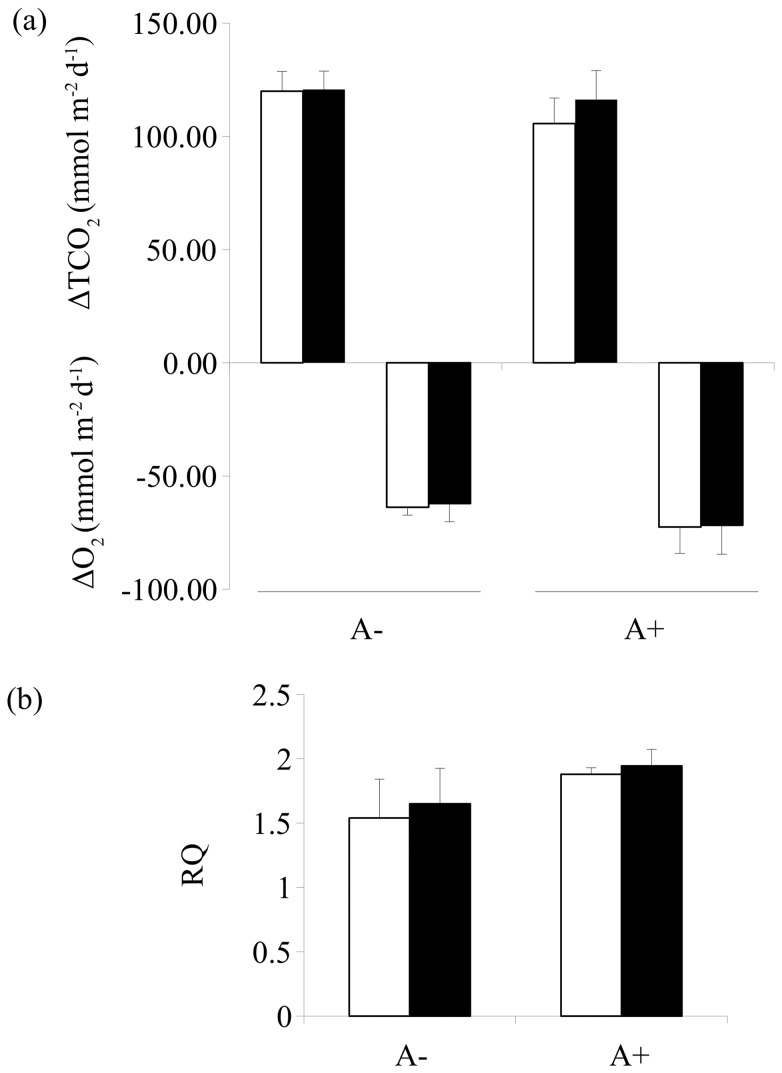
Mean (+1SE) for (a) benthic oxygen (O_2_) consumption and total carbon dioxide (TCO_2_) released during 12 hours dark incubation; (b) Respiratory quotient (RQ). In the graphs, A−: *A. marina* excluded; A+: procedural control for burial; the legend indicate the levels of detrital addition (0: no addition; 200∶200 g WW were added twice at a 1 week interval; 600∶600 g WW were added twice with a 1 week interval). Data were sampled one month after detrital addition.

Both *A. marina* exclusion and detrital enrichment or their interaction did not significantly change sediment-water exchanges of TCO_2_ and O_2_ nor the RQ respiration coefficient (TCO_2_:O_2_ ratio) (Table 2; Fig. 4).

**Table 2:** Two-way analyses of variance for sediment-water exchanges of O_2_ and TCO_2_ and for the benthic respiratory coefficient RQ.

		O_2_		TCO_2_		RQ	
	df	MS	F	MS	F	MS	F
Exclusion = E	1	8.8 10^−6^	2.38	252.08	0.78	0.29	2.39
Detrital enrichment = D	1	8.0 10^−9^	0.00	102.08	0.31	0.02	0.18
E×D	1	4.38 1^−7^	0.11*	70.08	0.22*	0.00	0.01*
Residual	8	4.10 1^−6^		325.08		0.14	

Significant values (*P*<0.05) are in bold. Data were not-transformed (*P*_C-cochran_ >0.05), except for O_2,_ which was arc-tangent transformed to homogenise the residual variances. Asterisk indicates pooling of interaction when *P*>0.25.

### Microalgal Biomass and Macrofauna

**Figure 5:**
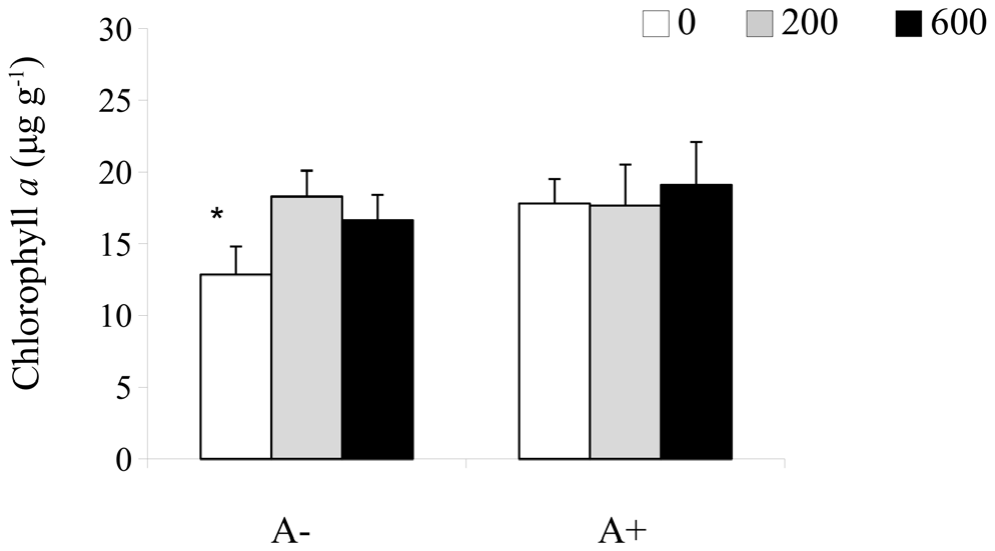
Mean (+1SE) for chlorophyll *a* concentration at the surface sediment (1 cm depth). In the graphs, A−: *A. marina* excluded; A+: procedural control for burial; the legend indicate the levels of detrital addition (0: no addition; 200∶200 g WW were added twice at a 1 week interval; 600∶600 g WW were added twice with a 1 week interval). Data were sampled one month after detrital addition. Asterisk indicates significant differences.

There was a significant interaction between *A. marina* exclusion and detrital enrichment for microalgal biomass (Table 3; Fig. 5). Lugworm exclusion had a negative effect on microalgal biomass in the non-enriched plots (SNK test: 0: A+>A−). In the lugworm-excluded sediment, detrital enrichment enhanced microalgal biomass (SNK test: A−: 0<200 = 600). Values were similar to those measured for the A+ control plots (treatments 200 and 600 in Fig. 5).

**Table 3:** Two-way analyses of variance for chlorophyll *a,* macrofauna abundance and number (N) of species.

		Chlorophyll a		Abundance		N of species	
	df	MS	F	MS	F	MS	F
Exclusion = E	1	72.23	1.27	0.48	0.69	0.03	0.00
Detrital enrichment = D	2	53.74	**4.02**	0.29	0.56	30.00	**7.11**
E×D	2	56.80	**4.25**	2.34	**4.81**	17.73	**4.21**
Residual	24	13.37		0.49		4.22	

Significant values (*P*<0.05) are in bold. Data were sqrt-transformed for macrofauna abundance.

**Figure 6:**
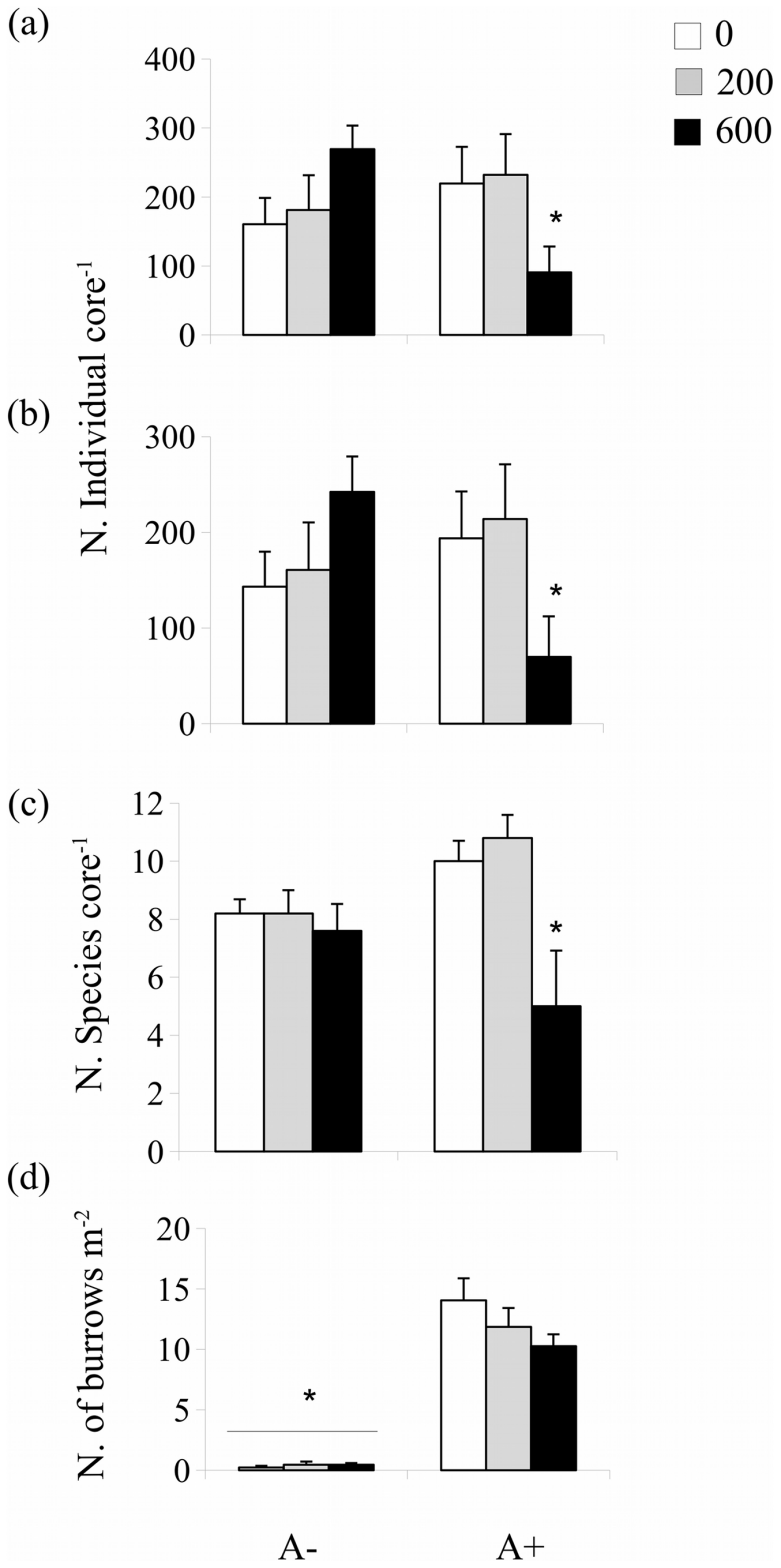
Mean (+1SE) for (a) total number of macrofauna individuals, (b) abundance of *Hydrobia ulvae*, (c) number of macrofauna species and (d) numbers of *Arenicola marina* burrows in the plots where the mosquito net was buried (A−) and in the procedural control plots (A+) at the end of the experiment, e.g; 1 month from the second addition of detrital *Ulva* (0: no addition; 200∶200 g WW were added twice at a 1 week interval; 600∶600 g WW were added twice with a 1 week interval). Data were sampled one month after detrital addition. Asterisk indicates significant differences.

A significant interaction between *A. marina* exclusion and detrital enrichment was also found for macrofauna abundance and number of species (Table 3). Abundance was lowered by the highest level of detrital enrichment in the plots where *A. marina* was present (Fig. 6a; SNK test: A+: 0 = 200>600). Overall, both the dominant gastropod *Hydrobia ulvae* and all other remaining macrofauna decreased in abundance, especially nereids, spionids and bivalves (ANOVA: F_2, 24_ = 4.26, *P* = 0.026 for *H. ulvae,* Fig. 6b; F_2, 24_ = 4.65, *P* = 0.019 for remaining taxa, data were square-root transformed; SNK tests for both: A+: 0 = 200>600). Number of species showed a similar pattern, decreasing in response to the highest level of enrichment in the A+ plots (Fi. 6C; SNK test: A+: 0 = 200>600). Detrital enrichment seemed to not affect the abundance of the lugworms as the number of *Arenicola* burrows remained relatively stable at different levels of detrital enrichment (2 way ANOVA: *F*_2, 12_ = 0.88, *P* = 0.34, Fig. 6d).

### The Fate of Detrital Carbon

**Figure 7:**
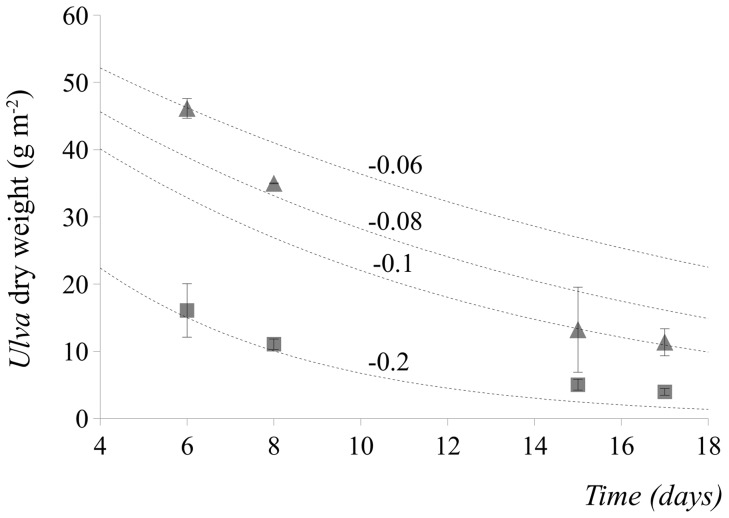
Decomposing curves for *Ulva* detritus in lugworm excluded (treatment A−; square symbols in the graphs) or procedural control sediments (treatment A+; triangle symbols in the graph). Symbols indicate mean (± SE) for macroalgal biomass and are relative to experiments 2.

**Figure 8:**
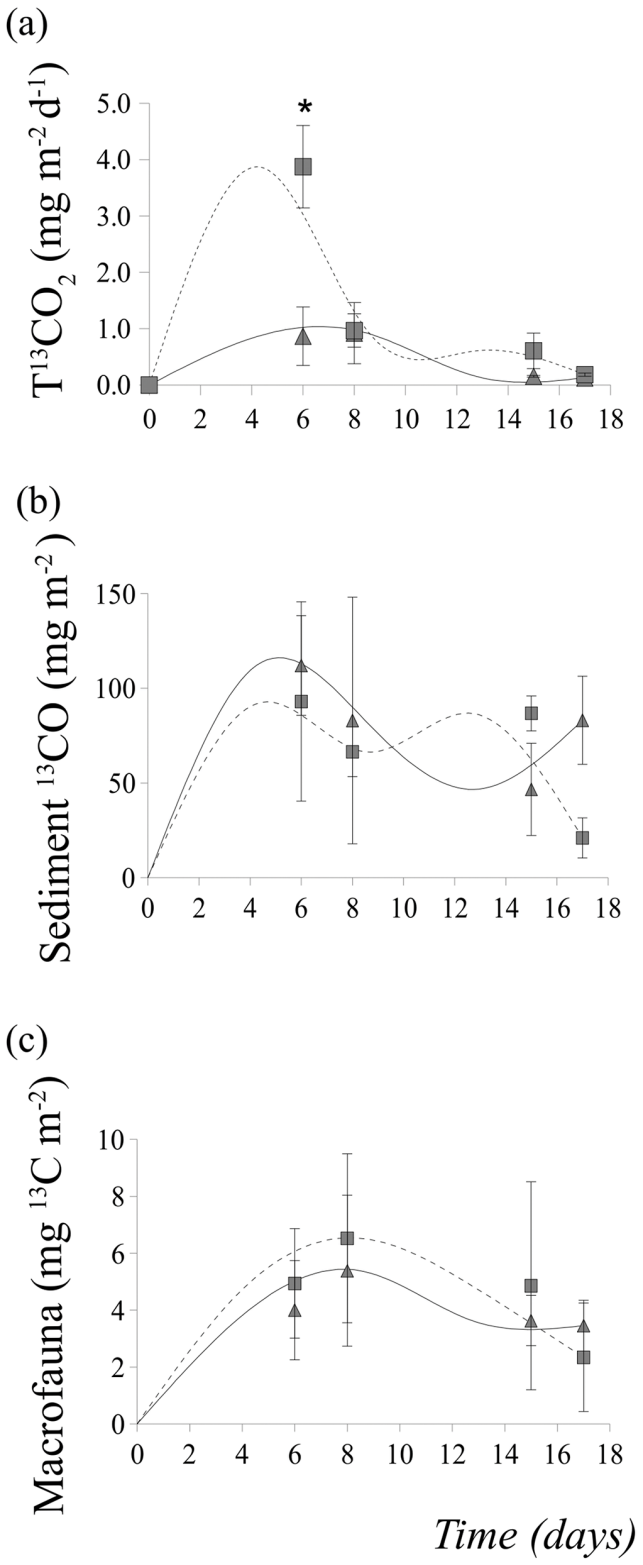
Mean ± SE for (a) T^13^CO_2_ released from the sediment to the water column (sum of dry and wet fluxes); (b) amount of ^13^C organic carbon in the bulk sediment (averaged over depth profile) and (c) amount of ^13^C carbon incorporated by macrofauna. Data are fitted to the LOESS smoothing with an interval of 4 and 1 polynomial degree. Values are averaged over the two plots where *A. marina* was excluded (treatment A−; square symbols in the graphs) and the two procedural control plots (treatment A+; triangle symbols in the graph). Data refers to the experiment 2. Asterisk indicates significant differences.

There were differences in the rate of *Ulva* decomposition following *A. marina* exclusion. The decomposition constant k was −0.2 for the detritus added to the *A. marina* excluded sediment (A− plots in Fig. 7). Interestingly, the A+ plots did not fit well the exponential curve using the same constant k through the decomposition. Rather, at the beginning of decomposition, they fitted k values between −0.06 and −0.08, whereas at the end they fitted a K constant of −0.1 (Fig. 7). There was a significant interaction between exclusion treatment and time after detrital enrichment for the flux rate of T^13^CO_2_ from the sediment to the water column (Table 4). More T^13^CO_2_ was released by the sediment in the exclusion treatment within 6 days from burial (Fig. 8a). Respiration rates were (mean ± SE) 0.84±0.25 and 0.12±0.03 mg ^13^C m^−2^ day^−1^ for dry and wet fluxes, respectively.

**Table 4:** Repeated measure analyses of variance and sphericity tests for the ^13^C incorporation in macrofauna and bulk sediment, and for the ^13^CO_2_ released from the benthos (benthic respiration) during detrital decomposition at an intermediate level of detrital enrichment (200 gWW plot^−1^added twice).

		Macrofauna		Bulk sediment		Benthic respiration	
	df	MS	F	MS	F	MS	F
Exclusion = E	1	5.97 10^−7^	0.12	4.27 10^−4^	0.22	1.52 10^−5^	**9.73**
Time = T	3	3.13 10^−7^	0.60	8.78 10^−4^	1.02	1.84 10^−5^	**14.45**
Residual (Plots)	2	4.96 10^−6^		1.96 10^−3^		1.56 10^−6^	
							
T×E	3	6.21 10^−7^	0.12	8.79 10^−4^	1.02	8.98 10^−6^	**7.07**
Residual (T×Plots)	6	5.21 10^−6^		8.59 10^−4^		1.27 10^−6^	
Sphericity test (χ^2^) n = 6		7.80		4.83		8.06	

Significant values (P<0.05) are in bold. Sphericity test was not significant.

Part of the ^13^C remained in the benthos and it was incorporated in the bulk sediment and in the macrofauna. The amount incorporated was variable among plots, but it did not significantly vary between exclusion treatments or time (Table 4; Fig. 8 b–c). *H. ulvae* incorporated the largest amount of ^13^C among all macrofauna taxa (mean ± SE: 3.2±0.8 and 1.2±0.4 mg ^13^C m^−2^, for *H. ulvae* and remaining fauna, respectively).

## Discussion

Bioturbation can enhance solute penetration in deeper sediments, their exchange between the sediment and the overlying water and re-distribute organic material along the vertical profile [36], [37], [41]. As such, the loss of species that are important contributors to bioturbation may greatly change the response to detrital decomposition of sediment biogeochemistry. In laboratory experiments, for instance, *A. marina* and the polychaete *Nereis diversicolor* were found to increase turnover of carbon and nutrients during detrital macroalgal decomposition [19], [36]. The exclusion of *A. marina* from intertidal sediments was thus expected to modify the biogeochemical response to macroalgal detrital enrichment. Conversely, at the end of decomposition (1 month after the second enrichment) benthic respiration (O_2_ and TCO_2_ sediment-water exchanges) and sediment organic carbon content did not change as an effect of lugworm exclusion.

*Ulva* amount and time of sampling (one month after enrichment) allowed decomposition and release of a large quantity of organic matter, which could be mineralized or accumulated in the sediment, thereby modifying sediment organic carbon, inorganic carbon dissolved in the interstitial water and fluxes at the sediment-water interface. *Ulva* halftime decomposition rate is within 15 days [30]. By considering the range of k constant calculated for this study (between −0.06 and −0.2), at least 85% *Ulva* would be decomposed at the time of sampling for both enrichment levels. If one considers that dried sediment weighs about 1600 g l^−1^ and *Ulva* organic carbon content is around 40% of dried tissues [34], this decomposed detritus corresponds to organic carbon concentrations ranging between 0.15 and 1.2% sediment dry weight for the top first cm, roughly 2–10 times the concentration of organic carbon found in natural sediments for low and high enrichment, respectively (see the non-enriched sediment in Fig. 2a). The lack of sediment carbon accumulation and changes in TCO_2_ fluxes at the sediment-water interface indicated that the benthic system was able to remove the carbon derived from *in situ* decomposition and that bioturbation did not play a central role in detrital carbon recycling.

It is important to re-iterate that fluxes were measured during 12 h dark incubation of sediment cores without lugworms, which indicated the overall indirect effect of lugworms on detrital benthic mineralization, but did not include any direct effects of lugworms due to burrow ventilation during the period of incubation. The importance of ventilation was instead assessed in the field by measuring porewater DIC, which increased after lugworm removal, indicating a decreased solute exchange due to *A. marina* loss. Interestingly, these changes were variable along the vertical sediment profile and were offset by the addition of detrital macroalgae, which supported the idea that fast mineralization of detrital carbon in these sediments is only in part due to bioturbation. The complexity of the relationships between species, functions and response to disturbance can increase with increasing ecological realism [15], [46]. Our experiments were done *in situ* and they were designed to test effects of *A. marina* under natural environmental conditions. Detrital macroalgae were deliberately added to the sediment, without using artificial nets that could limit detrital loss. This method exposed decomposing detritus to tides and waves, especially when detritus remained at the sediment surface. The role of bioturbation on sediment biogeochemistry has been mainly assessed through laboratory studies. The few field studies on the role of *Arenicola* on porewater, fluxes and organic matter decomposition have revealed that its role may vary and decrease considerably in nature [19], [37], [47]. By comparing field to laboratory experiments, it was found, for instance, that solute exchange was more variable in the field because it was not only ruled by bioturbation but also by advection related to waves and tides and by differences in the reactivity of sediment organic matter related to the complexity of biological communities of primary producers and consumers [47]. This is particularly true when experiments are done in permeable sediment, where *A. marina* is often a dominant species [48]. In these sediments, hydrodynamics can enhance advective flow from the porewater to the overlying water column, rule the exchange of solutes and organic matter at the sediment-water interface and overwhelm the biological effect of bioturbation [49]. Although our experiments were done in a relatively sheltered sand flat, as indicated by the dominance of fine-grained sand, numerous ripple marks were visible at low tide (Fig. 2) and they probably indicated strong hydrodynamics generated by waves and tides. In these sediments, hydrodynamics could accelerate the exchange of solutes produced during decomposition, the removal of macroalgal detritus from the sediment and override the effect of bioturbation on the response of sediment biogeochemistry to detrital macroalgae.

The decomposition rate of *Ulva* in these sediments was fast, as indicated by the estimated k constants (k = −0.2 and −0.1 for A− and A+, respectively; Fig. 7). These values were at the limit or even exceeded the range of values typical of *Ulva* detritus, which varies between −0.04 and −0.1 [34]. More importantly, the estimated k constant for the lugworm-exclusion sediment was twice that of the control plots. In addition, the biomass of macroalgal debris was, on average, 6 times larger in controls than in lugworm-excluded sediments (2 and 12 g DW per plot^−1^ for A+ and A−, respectively). This may bring to the conclusion that despite hydrodynamics could play an important role in determining benthic response to detrital macroalgae, *A. marina* could represent an important sink for detritus and it could modify *in situ* decomposition rate, as found for other burrowing bioturbators [39], [50]. These species can mechanically bury macroalgae in their funnels, thereby decreasing detritus availability to surface sediment and slowing down decomposition rate, while maintaining a pool of organic matter in the sediment. Buried detritus and slowed decomposition rate could, in turn, modify mineralization processes. In this study, a peak in T^13^CO_2_ release to the water column was identified at an early stage of decomposition in *A. marina*-excluded sediment plots, suggesting that the loss of *A. marina* accelerated the mineralization of detrital *Ulva* at an early stage of decomposition. This explanation of increased mineralization following *A. marina* loss was supported by the finding that porewater DIC decreased in response to detrital addition in *A. marina* excluded sediments. In addition, simultaneously to this decrease, macroalgal biomass increased. Probably, without the mechanical action of burying macroalgal fragments by *Arenicola*, detrital macroalgae remaining on sediment surface and decomposing faster released nutrients that enhanced bacterial metabolism and their carbon consumption. Evidence for increased carbon consumption during biomass decomposition has been found for plant litter in freshwater ecosystems [51], [52]. In addition, benthic microalgae are often regulated by bottom-up processes [53] and they could greatly benefit from nutrient release, thereby contributing to recycling the mineralized detrital carbon. Interestingly, detrital enrichment and lugworms had similar effects on microalgal biomass. Probably, both lugworm bioirrigation and detrital decomposition can provide nutrients necessary to regulate microalgal growth [19], [28], [37], [47].

*A. marina* is believed to influence the assembly rules and the distribution of macrofauna and, as such, their patterns of response to disturbances by altering the sedimentary habitat (e. g. [37], [41]). Our results showed a negative effect of *A. marina* on macrofauna response to detrital addition since both macrofauna abundance and diversity decreased following the highest level of enrichment in presence of lugworms. The increased detrital burial by *A. marina* could play a central role in determining this response. Patches of detrital macroalgae in sediments can govern the spatial and temporal variability of macrofauna patterns of distribution. The presence of detritus may decrease numbers of individuals and diversity because of hypoxia during decomposition. Once detritus has decomposed, fast recolonizing taxa can promptly occupy the previously disturbed patches and benefit from increased food supply [29]. Detrital burial due to lugworm activity probably prolonged hypoxic conditions and slowed down macrofauna recovery. The decrease in abundance common to all taxa and the loss of diversity could corroborate this explanation as anoxic conditions related to detrital decomposition deplete most taxa abundance and diversity [29]. Moreover, burial could decrease detrital availability to surface consumers and therefore prevent their colonization, once hypoxia ceases. Macroalgal supply to sediment surface can represent an important source of food for surface grazers and detritivores, such as the gastropod *Hydrobia ulvae* [34], [54]. Part of detrital carbon was transferred to macrofauna, especially to *H. ulvae* (experiment 2, Fig. 7c), which was the numerically dominant species in these area. Its important role for detrital carbon transport to the food web was found previously in the surrounding area [34]. This species is highly mobile and it crawls on sediment surface for feeding. Its local abundance might thus vary rapidly following food availability on surface sediment.

In summary, our field experiment showed that *A. marina* can be an important sink for detrital *Ulva.* However, conversely to what it should be expected based on current research knowledge, this species has moderate, sometimes negative effects on the response to detrital enrichment of sediment biogeochemistry and macrofauna. *A. marina* loss causes the sedimentary ecosystem to become a faster recycler of detrital macroalgae, enhancing detrital loss, carbon consumption and mineralization. This may bring to the conclusion that the loss of this species might be unimportant or somewhat beneficial for certain intertidal ecosystems. However, fast recycling and detrital dispersal could undermine carbon storage capacity of benthos and, in turn, the stability of whole estuarine ecosystem facing organic enrichment and eutrophication.

These findings clearly demonstrate that under natural conditions, the local extinction of an ecosystem engineer may have complex effects on the ecological stability of marine sediment biogeochemistry and benthic communities of consumers. They also highlight the importance of performing experiments under natural conditions if we are to understand and predict the response of ecosystems to perturbation and species loss.
